# Acquisition of a Lexicon for Family History Information: Bidirectional Encoder Representations From Transformers–Assisted Sublanguage Analysis

**DOI:** 10.2196/48072

**Published:** 2023-06-27

**Authors:** Liwei Wang, Huan He, Andrew Wen, Sungrim Moon, Sunyang Fu, Kevin J Peterson, Xuguang Ai, Sijia Liu, Ramakanth Kavuluru, Hongfang Liu

**Affiliations:** 1 Department of Artificial Intelligence and Informatics Mayo Clinic Rochester, MN United States; 2 Center for Digital Health Mayo Clinic Rochester, MN United States; 3 Department of Computer Science University of Kentucky Lexington, KY United States; 4 Division of Biomedical Informatics Department of Internal Medicine University of Kentucky Lexington, KY United States

**Keywords:** electronic health record, natural language processing, family history, sublanguage analysis, rule-based system, deep learning

## Abstract

**Background:**

A patient’s family history (FH) information significantly influences downstream clinical care. Despite this importance, there is no standardized method to capture FH information in electronic health records and a substantial portion of FH information is frequently embedded in clinical notes. This renders FH information difficult to use in downstream data analytics or clinical decision support applications. To address this issue, a natural language processing system capable of extracting and normalizing FH information can be used.

**Objective:**

In this study, we aimed to construct an FH lexical resource for information extraction and normalization.

**Methods:**

We exploited a transformer-based method to construct an FH lexical resource leveraging a corpus consisting of clinical notes generated as part of primary care. The usability of the lexicon was demonstrated through the development of a rule-based FH system that extracts FH entities and relations as specified in previous FH challenges. We also experimented with a deep learning–based FH system for FH information extraction. Previous FH challenge data sets were used for evaluation.

**Results:**

The resulting lexicon contains 33,603 lexicon entries normalized to 6408 concept unique identifiers of the Unified Medical Language System and 15,126 codes of the Systematized Nomenclature of Medicine Clinical Terms, with an average number of 5.4 variants per concept. The performance evaluation demonstrated that the rule-based FH system achieved reasonable performance. The combination of the rule-based FH system with a state-of-the-art deep learning–based FH system can improve the recall of FH information evaluated using the BioCreative/N2C2 FH challenge data set, with the F1 score varied but comparable.

**Conclusions:**

The resulting lexicon and rule-based FH system are freely available through the Open Health Natural Language Processing GitHub.

## Introduction

Family history (FH) has long been regarded as a core element in caring for patients who have varied health concerns [1], with the capability to significantly enhance the delivery of precision medicine [2]. However, FH data are underused for actionable risk assessment [1]. One barrier to using FH information is provider preference in recording the collected FH information in an unstructured format (eg, clinical notes) [3] as opposed to within electronic health record (EHR) structured data [4]. As clinical text tends to be unstructured, the information contained within is computationally inaccessible relative to that contained in structured records. This lack of computational accessibilities poses a challenge in using FH information for downstream data analytics or clinical practice (eg, via clinical decision support). One approach to render information data computationally accessible is through the use of natural language processing (NLP), thus motivating our work to develop an NLP system that can extract and normalize FH information.

Despite the majority of clinical NLP measurement studies focusing on statistical approaches, rule-based NLP systems based on semantic lexicons and rule patterns are popular among observational studies [5] for cancer research and practice [6,7]. With the advantages of ensuring process transparency, implementability, and scientific rigor, semantic lexicons and rule patterns are interpretable and easily modifiable, conforming to the FAIR (Findable, Accessible, Interoperable, and Reusable) and RITE (Reproducible, Implementable, Transparent, and Explainable) principles [8,9] for scientific data management. In addition, semantic lexicons and rule patterns capture sublanguage characteristics of domains that can be portable and generalizable to other applications [10]. By sublanguage, we refer to domain-specific linguistic and lexical patterns that are more prominent in free text in specialized fields such as medicine.

One popular lexical resource for clinical NLP is the Unified Medical Language System (UMLS), a repository of biomedical vocabularies distributed by the US National Library of Medicine, integrating over 200 biomedical vocabularies. A source vocabulary contained within the UMLS is the Systematized Nomenclature of Medicine Clinical Terms (SNOMED-CT), which is the recommended coding system for clinical problems. As not all terms in the UMLS or SNOMED-CT are part of the FH sublanguage, in this study, we exploited a corpus-driven method with pretrained language models to build an FH semantic lexicon with the normalization feature and reasonable size and coverage.

There have been previous efforts focused on creating semantic lexicons for clinical NLP. Johnson [11] automatically constructed a semantic lexicon based on the Specialist Lexicon of the UMLS, which can assist NLP analysis of a medical narrative with the semantic preference options of selecting semantic type. Luo et al [12] created a semantic lexicon using UMLS knowledge sources by leveraging a corpus from ClinicalTrials.gov. Liu et al [13] constructed a corpus-driven semantic lexicon based on the UMLS assisted by variants mined and usage information gathered from clinical text.

Regarding deep learning–based approaches, pretrained language models such as bidirectional encoder representations from transformers (BERT) [14] can learn the structure of language (ie, the basic semantic and syntax information) through unsupervised training on a large corpus of unlabeled text [15]. Given a new task, such pretrained models can be fine-tuned with a small number of annotated samples to perform well [16].

With respect to the FH information extraction task, we hypothesized that terms in a large-scale corpus having semantic types similar to the entities labeled in the data set used for fine tuning can be detected by fine-tuned BERT models in a named entity recognition task. Additionally, we hypothesized that corpus-driven methods would enable more term variants to be discovered from real-world EHR data, from which the lexicon results could further enhance and empower FH information extraction systems. Operating under these two hypotheses, we here present an FH lexicon derived through a combination of these two approaches. To demonstrate the usability of the FH lexicon, we further developed a rule-based FH system based on the lexicon that extracts FH entities and relations specified in previous FH challenges and evaluated its performance accordingly. In terms of system development, we consider that an FH system that prioritizes recall is highly desired for NLP-assisted curation in EHR-based studies.

## Methods

### FH Concepts

**Figure 1:**
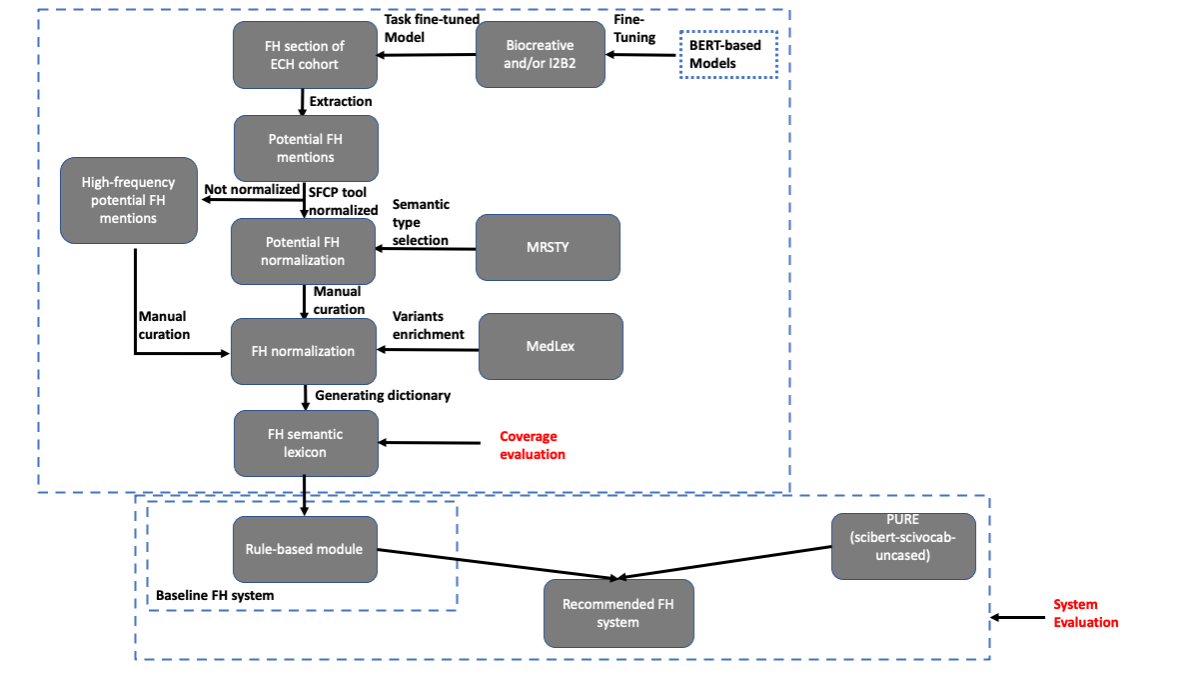
Study design. BERT: bidirectional encoder representations from transformers; ECH: employee and community health; FH: family history; I2B2: 2012 i2b2 natural language processing challenge data set (training data set); PURE: Princeton University Relation Extraction; SFCP: standardization framework for clinical problems.

Before assembling an FH lexicon, we defined FH concepts as those belonging to the selected UMLS semantic types within the “DISO” (disorder) semantic group (Table 1), excluding T050 (experimental model of disease) [17]. Figure 1 shows our study design. We first fine-tuned Bio_ClinicalBERT, UmlsBERT, and bert-base-uncased models, and selected the model with the best performance. We then used the selected model to extract potential disorder/finding related mentions in a large clinical corpus. Subsequently, potential FH mentions were automatically normalized, manually curated, and prepared into symbolic lexicon format compatible with the Open Health Natural Language Processing (OHNLP) Toolkit’s NLP engine MedTagger [18]. A coverage evaluation was conducted for the lexicon. To demonstrate the usability of the lexicon, a rule-based FH system was developed to extract FH information. In parallel, we also experimented with a deep learning–based FH system for FH information extraction. Previous FH challenge data sets were used for evaluation [19,20].

**Table 1:** Selected semantic types as family history concepts.

Semantic type code	Semantic type term
T019	Congenital abnormality
T020	Acquired abnormality
T037	Injury or poisoning
T047	Disease or syndrome
T048	Mental or behavioral dysfunction
T049	Cell or molecular dysfunction
T190	Anatomical abnormality
T191	Neoplastic process
T033	Finding
T046	Pathologic function
T184	Sign or symptom

### Resources

#### Overview

Here, we introduce the resources used for lexicon construction and all relevant evaluations. Specifically, the 2018 BioCreative FH challenge training set and the 2012 I2B2 training set were used as the supervised data sets to fine-tune the deep learning models for lexicon preparation. Various data sets were experimented with based on a hypothesis that more data could encompass more semantic contexts of potential FH mentions. The 2018 test set was used to evaluate lexicon coverage. The 2018 and 2019 FH challenge training sets were used for training of the rule-based FH system and fine-tuning the deep learning–based FH system, and the 2018 and 2019 FH challenge test sets were used for evaluating the performance of the FH systems in extracting FH entities (task 1) and relations (task 2). A large EHR corpus was used for collecting potential FH mentions. MedLex was used to further enrich term variants of FH concepts extracted by the selected model. The UMLS was used for semantic type selection, while UMLS and SNOMED-CT were used for a comparison of size with our corpus-driven dictionary. Table 2 summarizes specific applications of the resources in the lexicon construction and all relevant evaluations. Detailed descriptions for each resource are provided below.

**Table 2:** Specific applications of resources in the lexicon construction and evaluations.

Application		A^a^	B^b^		C^c^	D^d^	E^e^	A+E	EHR^f^ corpus	MedLex	UMLS^g^	SNOMED-CT^h^
**Lexicon construction**												
	Fine-tuning the BERT^i^ model	✓					✓	✓				
	Dictionary entry collection								✓			
	Dictionary concept enrichment									✓		
	Semantic type selection										✓	
**FH^j^ system development**												
	Development of rule-based FH system	✓	✓									
	Fine-tuning deep learning–based FH system	✓	✓									
**Evaluation**												
	Dictionary coverage			✓							✓	✓
	Challenge task 1			✓		✓						
	Challenge task 2			✓		✓						

^a^A: Training set (BioCreative).

^b^B: Training set (N2C2).

^c^C: Testing set (BioCreative).

^d^D: Testing set (N2C2).

^e^E: Training set (I2B2/2010).

^f^EHR: electronic health record.

^g^UMLS: Unified Medical Language System.

^h^SNOMED-CT: Systematized Nomenclature of Medicine Clinical Terms.

^i^BERT: bidirectional encoder representations from transformers.

^j^FH: family history.

#### Synthetic FH Annotation Data Sets (A-D)

As the organizer of the BioCreative/OHNLP 2018 Family History Extraction Task [19] and 2019 NLP Clinical Challenge (N2C2)/OHNLP shared task [20], we curated deidentified annotation data sets based on synthetic clinical narratives. Data set A corresponds to data set C in the BioCreative Challenge and data set B corresponds to data set D in the N2C2 Challenge. FH was annotated as “observation” and defined as any health-related problem, including diseases, smoking, suicide, and drinking, while excluding auto accidents, surgeries, and medications [19]. Family members (FMs), observation, age, and living status were annotated as entities, and then all entities related to an FM category were linked into one chain. We further enhanced the resulting annotations in the data sets by normalizing observations to SNOMED-CT codes and correcting errors in previous annotations. The reannotated data sets are accessible based on the Data Use Agreement. Multimedia Appendix 1 shows the statistical comparison between original and enhanced annotations.

#### I2B2 Data Set (D)

The 2012 I2B2 NLP challenge organizers provided a fully deidentified data set with annotations for temporal relations as well as those generated from previous challenges such as the 2010 challenge of clinical concept extraction (problems, tests, treatments) [21], where problems include symptoms, complaints, diseases, and diagnoses.

#### EHR Raw Corpus

The raw corpus used in the study consists of 9,426,352 text segments extracted from the Family History section of clinical notes prior to 2013 of a primary care cohort (ie, the employee and community health cohort), which contains 83,000 patients at Mayo Clinic.

#### Dictionary Resources

MedLex is a semantic lexicon built on a large corpus of clinical documents collected at Mayo Clinic and from the UMLS (2011AA version) [13]. MedLex contains term variants from real-world EHRs, serving as a practical dictionary resource for FH lexicon expansion. We used MedLex to further enrich term variants of FH concepts extracted by the selected model. The UMLS (2021AA) and SNOMEDCT_US concepts accessible through the UMLS were restricted to only English entries. The MRCONSO table of the UMLS, which includes over 200 source vocabularies, was used for coverage evaluation, and the MRSTY table of the UMLS was used for screening semantic types for each concept unique identifier (CUI) in the MRCONSO table.

### Ethical Approval

Use of the EHR raw corpus data was approved by the Mayo Clinic Institutional Review Board (17-003030) for Human Subject Research.

### BERT-Based Corpus Analysis for Lexicon Construction

#### BERT Models for Extraction of Potential FH Mentions

BERT-base-uncased was pretrained on an unsupervised NLP data set using a masked language modeling approach [14]. Bio_ClinicalBERT was initialized from BioBERT and trained on all Medical Information Mart for Intensive Care notes [22]. UmlsBERT is a contextual embedding model that integrates domain knowledge during the pretraining process via a novel knowledge augmentation through the UMLS Metathesaurus [23]. UmlsBERT and Bio_ClinicalBERT are domains related to this study, while BERT-base-uncased could be used as a baseline comparison. We used configurations mostly consistent with the recommendations in the original release of the models. The maximum sequence length was set to 512, the batch size was set to 16, the total number of training epochs was set to 100, and the weight decay was set to 0.01. An early stopping method was used to determine the optimal number of epochs and to prevent overfitting. The train/test split was 80/20, where the “train” split was used for training and the “test” split was used for validation.

We fine-tuned these models on the BioCreative training data set, I2B2 training data set, as well as the combination of the BioCreative and I2B2 training data sets. We then selected the model with the best performance to extract any potential FH mentions in the raw corpus having coarse-grained semantic types similar to the entities labeled in the supervised data.

#### Normalization

The extracted FH mentions were automatically normalized through a standardization framework for clinical problems (SFCP) [24]. This framework converts free-text clinical problem descriptions into standardized forms based on the UMLS CUI corresponding to SNOMED-CT concepts and the Health Level 7 Fast Healthcare Interoperability Resources (FHIR)-based structured representations, including the codified problem and all relevant modifiers and context. The CUIs associated with the SNOMED-CT concept were used for coding. For example, for the mention “allergy-induced asthma,” the framework outputs “C0440102 | Various patch test substance” and “C0155877 | Allergic asthma.”

#### Manual Curation and Further Enrichment

We reviewed all normalized FH mentions and retained the main normalized problem concepts with semantic types corresponding to those previously selected. For example, for the mention “allergy-induced asthma,” the codified problem “C0155877 | Allergic asthma” was kept and “C0440102 | Various patch test substance” was removed from the final lexicon. In some cases, one BERT-extracted mention can be normalized to several individual concepts through the automatic standardization. We then enriched the FH lexicon by keeping all individual concepts and obtaining associated variants from MedLex.

In addition, we manually mapped high-frequency mentions that occurred across at least 20 patients that were not automatically standardized to corresponding CUIs.

### Rule-Based FH System

To demonstrate the capability of the FH lexicon in extracting FH relations, we further implemented a rule-based FH system by integrating rules for FM identification with the resulting lexicon. Multimedia Appendix 2 shows three degrees of consanguinity we aggregated into FM identification rules. FH relations were then extracted based on co-occurrence within a clause of one sentence or across three adjacent sentences if coreference existed, as indicated by keywords such as “he,” “she,” “none of them,” “her,” or “his,” while excluding relations between FMs of spouse and FH. We implemented this as an OHNLP Toolkit module with code available on GitHub [25]. The implementation provides several output formats, including FHIR-based output, with FM and FH standardization conforming to FHIR standardization. The final output of the rule-based FH system includes entities and relations. The entity output includes file name (which links to document references with patients’ ID), sentence ID, chunk ID, entity type, concept, and certainty. The relation output includes file name, FM, side of family, text of observation, and certainty. An option is also available to output this information to CSV, instead of FHIR, format. To set SNOMED-CT condition codes as the standard, a separate mapping file is required due to SNOMED-CT licensing restrictions.

### Deep Learning–Based FH System

As information extraction remains a challenging task, it is preferred to investigate what is the gain from deep learning–based models. Therefore, we further implemented a deep learning–based FH system as follows. Note that we experimented with fined-tuned models for two purposes in this study. The first was to fine-tune models for identifying and collecting potential FH mentions from clinical texts to build a dictionary, as described in the previous section. Here, the second purpose was to fine-tune models for information extraction to automatically identify FH entities and relations.

The Princeton University Relation Extraction (PURE) system is an approach where the entity model builds on span-level representations and the relation model builds on contextual representations specific to a given pair of spans. As this pipelined approach has been demonstrated to be extremely effective, we implemented PURE using scibert-scivocab-uncased as the base encoder and fine-tuned it based on the BioCreative and N2C2 training data [26].

### Evaluation

#### Overview

We conducted two evaluation studies, including (1) a coverage evaluation of the lexicon and (2) a comparison study of a lexicon-based module with a deep learning–based module for FH information extraction.

#### Lexicon Coverage

To the best of our knowledge, our lexicon is the first to incorporate a large number of text variants and concepts of FH. Therefore, we compared the resulting lexicon with the UMLS and SNOMED-CT in terms of the number of concepts under each semantic type. We analyzed the lexicon coverage by calculating the number of concepts under each semantic type. In addition, we calculated the number of concepts and variants covered by the corpus-driven lexicon run against the BioCreative testing data set relative to annotated gold standards. True positive (TP) rate based on a partial match, false negative (FN) rate, and recall (TP/[TP+FN]) at the concept level and variant level were calculated.

#### Performance of FH Information Extraction

We evaluated the utility of the lexicon in identifying mentions of FMs and their associated attributes (side of family) using the BioCreative testing set and the N2C2 testing sets (ie, task 1 of the challenges). Precision, recall, and F1-scores were calculated as the performance metrics of the lexicon-based module, the deep learning–based module, and both.

We also evaluated the rule-based FH system’s ability to identify relations between FMs, observations, and living status using the BioCreative testing set and N2C2 testing sets (ie, task 2 of the challenges). This task was different between the 2018 BioCreative and 2019 N2C2 challenges in that the latter added a certainty attribute (negated or nonnegated) into relation extraction. Three sets of precision, recall, and F1 values were separately calculated using varying setups: rule-based module only, deep learning–based module only, and a combination of both. Our evaluation scheme is the same as that applied in the 2018 BioCreative and 2019 N2C2 FH challenges. We performed a general error analysis to investigate error sources. In addition, to further investigate how much the lexicon contributes to system performance, we performed an ablation study and specific error analyses.

## Results

Multimedia Appendix 3 shows the performance of the BERT-base-uncased, UmlsBERT, and Bio_clinicalBERT models fine-tuned on the BioCreative training data set, I2B2 training set, and on the combination of the BioCreative training set with the I2B2 training set. As the model fine-tuned on the combination of the two data sets outperformed other models, we selected the combined model to extract potential FH mentions from the corpus.

There were 72,518 unique entities identified by the Bio_clinicalBERT model fine-tuned on the combination data set, of which 47,250 (65.16%) were automatically normalized to 10,579 CUIs through the standardization framework for clinical problems. We manually normalized 148 entities occurring across more than 20 patients that were not automatically normalized to CUIs. For example, “typediabetes” with a frequency of 3693 was normalized to C0011854 (diabetes). Note that spellings such as “typediabetes” found in the EHRs are most likely typos due to physicians’ writing, backend EHR note processing, or tokenization challenges. Therefore, manual normalization is important. After semantic type screening, manual curation, and MedLex enrichment, the final FH lexicon contained 33,351 dictionary entities normalized to 6177 CUIs and 15,126 SNOMED-CT codes, with an average of 5.4 variants for each concept. Table 3 shows the comparison of sizes of various lexicons. The corpus-driven lexicon was more light-weighted, with more variants per concept. This implies that implementation with the lexicon for NLP tasks would be easier and more efficient with the corpus-driven lexicon than with the SNOMED-CT and UMLS lexicons.

Lexicon coverage evaluation on the BioCreative testing data set showed that there are 137 TP entities corresponding to 128 concepts and there are 16 FN entities, of which 6 entities had no corresponding concepts in the FH lexicon and 10 entities had 10 corresponding concepts in the FH lexicon. Concept-level recall was 95.8% and variant-level recall was 89.5%. For the N2C2 testing set, there were 507 TP entities corresponding to 214 concepts and 62 FN entities, of which 33 entities had no corresponding concepts in the FH lexicon and 29 entities had 26 corresponding concepts in the FH lexicon. Concept-level recall was 87.9% and variant-level recall was 89.1%. Table 4 shows the comparison of numbers of semantic types of CUIs in various lexicons. It can be observed that the corpus-driven lexicon contains less concepts under each semantic type compared with the UMLS and SNOMED-CT lexicons.

Table 5 shows the performance of FH systems for subtasks 1 and 2 of the BioCreative and N2C2 challenge data sets (original and reannotated) categorized by the rule-based FH system only, deep learning–based FH system only, and a combination of the two. For task 1, the highest F1 score was 0.8766 from the deep learning–based model on the original BioCreative data set and was 0.8061 on the original N2C2 data set. For task 2, the highest F1 score was 0.6206 from the deep learning–based module on the original BioCreative dataset and was 0.5940 from the combined results on the original N2C2 data set. The rule-based FH system based on the corpus-driven lexicon produced lower F1 scores in contrast with the deep learning–based FH system for both tasks, but higher or comparable recall for task 1 and higher recall for task 2. The combined results had the highest recall compared with the rule-based FH system or the deep learning–based FH system for task 1, ranging from 0.8669 to 0.9475. The combined results also showed the highest recall (ranging from 0.7109 to 0.8370) and varied F1 scores (ranging from 0.4288 to 0.6142) for task 2.

**Table 3:** Comparison of the size of lexicons.

Lexicon	Concepts (CUI^a^)	Variants	Average number of variants per CUI
Corpus-driven lexicon	6177	33,351	5.40
SNOMED-CT^b^	412,027	1,349,838	3.28
UMLS^c^	4,440,279	9,569,507	2.16

^a^CUI: concept unique identifier.

^b^SNOMED-CT: Systematized Nomenclature of Medicine Clinical Terms.

^c^UMLS: Unified Medical Language System.

**Table 4:** Statistical summary for semantic types of concept unique identifiers (CUIs) in the lexicon.

Semantic type code	Semantic type term	Number of CUIs		
		Corpus-driven lexicon	UMLS^a^	SNOMED-CT^b^
T019	Congenital abnormality	61	11,237	7034
T020	Acquired abnormality	62	4326	2105
T037	Injury or poisoning	236	113,258	29,371
T047	Disease or syndrome	2645	113,780	45,946
T048	Mental or behavioral dysfunction	376	9152	3318
T049	Cell or molecular dysfunction	24	4175	583
T190	Anatomical abnormality	94	6943	1703
T191	Neoplastic process	866	43,532	11,054
T033	Finding	1018	309,971	44,547
T046	Pathologic function	403	27,601	9098
T184	Sign or symptom	389	14,167	2931

^a^UMLS: Unified Medical Language System.

^b^SNOMED-CT: Systematized Nomenclature of Medicine Clinical Terms.

**Table 5:** Evaluation results for family history (FH) information extraction.

Task and data set		Deep learning–based FH system				Rule-based FH system				Combined		
		Precision	Recall	F1	Precision		Recall	F1	Precision		Recall	F1
**Task 1 Original**												
	2018 testing	0.8819	0.8729	0.8766	0.7877		0.8619	0.8211	0.7857		0.9475	0.8469
	2019 testing	0.8271	0.7835	0.8061	0.7191		0.8408	0.7740	0.7104		0.9007	0.7835
**Task 1 Reannotation**												
	2018 testing	0.8830	0.8607	0.8709	0.7988		0.8294	0.8125	0.8081		0.9193	0.8486
	2019 testing	0.7860	0.7747	0.7806	0.7149		0.7980	0.7536	0.7041		0.8669	0.7643
**Task 2 Original**												
	2018 testing	0.7189	0.5464	0.6206	0.5413		0.6000	0.5673	0.5518		0.7237	0.6142
	2019 testing	0.6841	0.5109	0.5851	0.4089		0.5571	0.4716	0.4699		0.8370	0.5940
**Task 2 Reannotation**												
	2018 testing	0.6777	0.5307	0.5962	0.5596		0.5980	0.5769	0.5573		0.7109	0.6142
	2019 testing	0.3309	0.8168	0.4629	0.4003		0.5292	0.4548	0.3060		0.8150	0.4288

A general error analysis showed that errors could be divided into two major sources. First, varied definitions of FH between different subtasks represented a confounding factor. FH in the gold standards was defined as any health-related problem, including diseases, smoking, suicide, and drinking, excluding auto accident, surgery, and medications [19], whereas the definition in the FH lexicon is based on semantic types (Table 1). For example, mastectomy, a procedure, was annotated as an observation (FH) in the challenge data sets, which is not in the scope of our lexicon. Second, the intrinsic difficulty of FH relation extraction and its textual representations presented several obstacles, particularly with symbolic systems. The rule-based FH system used simple heuristic rules, and therefore it is difficult to handle complex relationships, especially when the subtask 2 of challenges includes multiple layers of relationships.

In the ablation study, for task 2 based on the original BioCreative test set, the precision, recall, and F1 score for observation only (excluding living status) were 0.5419, 0.6265, and 0.5783, respectively, representing a slight improvement compared with the corresponding values of 0.5413, 0.6000, and 0.5673 for both observation and living status. For task 2 based on the original N2C2 test set, the precision, recall, and F1 score were 0.3984, 0.7033, and 0.5449, respectively, for observation only (excluding living status); 0.4275, 0.6658, and 0.5444, respectively, for both observation and living status; and 0.3730, 0.6675, and 0.5169, respectively, for both observation and certainty. Although living status and certainty alone had little impact on the performance, the combination of observation, living status, and certainty resulted in significantly lower performance of 0.4089, 0.5571, and 0.4716 for precision, recall, and F1, respectively.

## Discussion

FH has its own sublanguage. However, some terms related to FH may not actually be used in practice or may be used very rarely, such as “ancestor,” “descendant,” or “genealogy.” For this reason, these terms may not appear in the lexicon. As FH is specifically related to blood relations (consanguinity), it relates to the patient themselves. Therefore, the FM of spouse should not be considered, and FH elements relating to a spouse (rather than the patient) should consequently not be extracted. The advantage of this operation is the consistency with definitions of FM, resulting in a list of FH of the relevant FMs. The disadvantage may be missing the spouse’s relatives and associated FH information. There are some social, behavioral, and environment factors shared in the same household, which also represent critical information. However, these are social determinants of health and not part of the blood relations.

Collecting lexicon entries can be defined as a named entity recognition task, which is an important task for identifying meaningful terms and multiword phrases in free text [27]. In this study, we fine-tuned several BERT-based models for the purpose of identifying potential FH mentions from an EHR corpus, leveraging various data sets for the purpose of providing more context for fine-tuning BERT-based models. Lexicon entries were collected from a large clinical EHR corpus, mitigating the problem of missing entities caused by limited amounts of data. The dictionary coverage evaluation showed that it covers a greater range of lexical variants and focuses primarily on clinical concepts typically reported as part of FH relative to a direct lexicon generated from the UMLS and SNOMED-CT. Our corpus-driven lexicon features FH definitions based on semantic types, concept normalization to UMLS and SNOMED-CT CUIs, and manual curation, with the potential to resolve semantic ambiguity and promote interoperability among various systems. The rule-based FH system also provides standard Health Level 7 FHIR output to foster interoperability.

FH relation extraction is more relevant for downstream analysis compared with entity extraction. In previous challenges, the F1 score was regarded as the most important metric for relation extraction evaluation. The highest F1 score obtained from challenges was 0.5708 in the BioCreative challenge [19] and was 0.681 in the N2C2 challenge [20]. However, it is not our aim to compete with previous studies in terms of F1 scores. As relation extraction is still a challenging task, an FH system that prioritizes recall is highly desired for NLP-assisted curation in EHR-based studies. Our evaluation results showed that the rule-based FH system on top of the corpus-driven lexicon produced higher recall than that obtained with the deep learning–based FH system. In addition, the combined results from both the rule-based module and the deep learning–based FH system resulted in the highest recall for relation extraction, ranging from 0.7109 to 0.8370, which were higher than the recall values obtained in any previous challenge results, ranging from 0.3732 to 0.6810.

Note that we did not observe higher performance when using the reannotated data as compared to the original data. There may be two underlying reasons for this. First, reannotated data have not been used for training the rule-based system. Second, reannotated data were obtained by a professional annotator with deep domain knowledge, which makes the information extraction task harder. In addition, we observed that performance on 2019 N2C2 FH challenge data was worse than that on the 2018 BioCreative FH challenge data. This is mainly because the 2019 N2C2 FH challenge added a certainty attribute (negated or nonnegated) into the relation extraction, which made the relation extraction task harder.

Our FH synthetic data sets used for training and testing were from real clinical sentences, for which the observations, FMs, and ethnicities are shuffled among the whole corpus using a heuristic deidentification process. The granularity of the synthetic FH data sets is the same as that of real FH data. In this study, we have not exhaustedly compared all BERT-related models. Theoretically, large language models–empowered knowledge engineering is sufficient for lexicon entry collection from a clinical corpus. Our focus in this study was to provide a corpus-driven lexicon resource that leads to a rule-based FH baseline system for high-throughput analysis, while doing so in a manner that promotes interpretability and explainability for downstream applications.

We recommend that a comprehensive FH system include both a rule-based module and a deep learning–based module to obtain higher recall, which could facilitate manual curation. Although only the rule-based FH system can output normalized concepts, output from a deep learning–based FH system can be a rich source to enrich the lexicon. In the future, we will repeat the SFCP normalization for the output from the deep learning–based FH system to consistently improve the FH lexicon and the rule-based FH system. Meanwhile, we will continue to manually review the BERT-extracted entities without automatic normalization with frequency under 20, and look into other data sources such as social media so as to expand more concepts and/or term variants for the current lexicon. In addition, we will engage the user community to continuously refine the lexicon. We also plan to update the lexicon and rule-based FH system yearly, which will be distributed through the same open-source repository on GitHub.

There are three limitations to this study. First, although a large corpus with 83,000 patients was used for collection of potential FH variants, there is still a possibility that the FH information is not well represented. In addition, as the lexicon was developed using a largely monoinstitutional data resource, the lexicon may not be generalizable in other institutions. Second, during the entity normalization, we simply adopted an existing standardization framework, as it was not a priority of this study to focus on standardization method development. Third, we arbitrarily set a frequency cutoff of 20 for entities that were not automatically normalized to include in our manual review. However, we realize that some entities with low frequency also have the potential to contribute to the lexicon entries, such as “rectal ca” with a frequency of 18 and “highcholesterol” with a frequency of 12.

In summary, we constructed a corpus-driven FH lexicon to serve as a language resource for FH information extraction. Standardization of concepts in the FH lexicon and the rule-based FH system foster interoperability. The resulting lexicon and the rule-based FH system are freely available as part of the OHNLP Toolkit ecosystem. In the future, we will continue to expand more concepts and/or term variants of the current lexicon, and will explore the incorporation of the system for data curation efforts needed in various EHR-based studies and applications.

## Appendix

Multimedia Appendix 1 Statistical comparison between original and enhanced annotations.

Multimedia Appendix 2 Degree of consanguinity.

Multimedia Appendix 3 Performance of various BERT models fine-tuned on different data sets.

## Abbreviations

BERT: bidirectional encoder representations from transformers
CUI: concept unique identifier
EHR: electronic health record
FAIR: Findable, Accessible, Interoperable, and Reusable
FH: family history
FHIR: Fast Healthcare Interoperability Resource
FM: family member
FN: false negative
N2C2: 2019 NLP Clinical Challenge
NLP: natural language processing
OHNLP: Open Health Natural Language Processing
PURE: Princeton University Relation Extraction
RITE: Reproducible, Implementable, Transparent, and Explainable
SFCP: standardization framework for clinical problems
SNOMED-CT: Systematized Nomenclature of Medicine Clinical Terms
TP: true positive
UMLS: Unified Medical Language System

## Data Availability

The source code underlying this article is available on GitHub [25].

## References

[1] Ginsburg GS, Wu RR, Orlando LA (2019). Family health history: underused for actionable risk assessment. Lancet.

[2] Bylstra Y, Lim WK, Kam S, Tham KW, Wu RR, Teo JX, Davila S, Kuan JL, Chan SH, Bertin N, Yang CX, Rozen S, Teh BT, Yeo KK, Cook SA, Jamuar SS, Ginsburg GS, Orlando LA, Tan P (2021). Family history assessment significantly enhances delivery of precision medicine in the genomics era. Genome Med.

[3] Friedlin J, McDonald CJ (2006). Using a natural language processing system to extract and code family history data from admission reports. AMIA Annu Symp Proc.

[4] Polubriaginof F, Tatonetti NP, Vawdrey DK (2015). An assessment of family history information captured in an electronic health record. AMIA Annu Symp Proc.

[5] Fu S, Wang L, Moon S, Zong N, He H, Pejaver V, Relevo R, Walden A, Haendel M, Chute CG, Liu H (2023). Recommended practices and ethical considerations for natural language processing-assisted observational research: A scoping review. Clin Transl Sci.

[6] Wang L, Fu S, Wen A, Ruan X, He H, Liu S, Moon S, Mai M, Riaz IB, Wang N, Yang P, Xu H, Warner JL, Liu H (2022). Assessment of electronic health record for cancer research and patient care through a scoping review of cancer natural language processing. JCO Clin Cancer Inform.

[7] Fu S, Chen D, He H, Liu S, Moon S, Peterson KJ, Shen F, Wang L, Wang Y, Wen A, Zhao Y, Sohn S, Liu H (2020). Clinical concept extraction: a methodology review. J Biomed Inform.

[8] Wilkinson MD, Dumontier M, Aalbersberg IJJ, Appleton G, Axton M, Baak A, Blomberg N, Boiten J, da Silva Santos LB, Bourne PE, Bouwman J, Brookes AJ, Clark T, Crosas M, Dillo I, Dumon O, Edmunds S, Evelo CT, Finkers R, Gonzalez-Beltran A, Gray AJG, Groth P, Goble C, Grethe JS, Heringa J, 't Hoen PAC, Hooft R, Kuhn T, Kok R, Kok J, Lusher SJ, Martone ME, Mons A, Packer AL, Persson B, Rocca-Serra P, Roos M, van Schaik R, Sansone S, Schultes E, Sengstag T, Slater T, Strawn G, Swertz MA, Thompson M, van der Lei J, van Mulligen E, Velterop J, Waagmeester A, Wittenburg P, Wolstencroft K, Zhao J, Mons B (2016). The FAIR Guiding Principles for scientific data management and stewardship. Sci Data.

[9] Fu S (2021). TRUST: Clinical text retrieval and use towards scientific rigor and transparent process. Thesis. University of Minnesota.

[10] Moon S, He H, Liu H (2022). Sublanguage characteristics of clinical documents.

[11] Johnson SB (1999). A semantic lexicon for medical language processing. J Am Med Inform Assoc.

[12] Luo Z, Duffy R, Johnson S, Weng C (2010). Corpus-based approach to creating a semantic lexicon for clinical research eligibility criteria from UMLS. Summit Transl Bioinform.

[13] Liu H, Wu ST, Li D, Jonnalagadda S, Sohn S, Wagholikar K, Haug PJ, Huff SM, Chute CG (2012). Towards a semantic lexicon for clinical natural language processing. AMIA Annu Symp Proc.

[14] Devlin J, Chang M, Lee K, Toutanova K (2018). Bert: Pre-training of deep bidirectional transformers for language understanding. arXiv.

[15] Clark K, Khandelwal U, Levy O, Manning C (2019). What does BERT look at? an analysis of BERT's attention. arXiv.

[16] Min S, Seo M, Hajishirzi H (2017). Question answering through transfer learning from large fine-grained supervision data. arXiv.

[17] Bodenreider O, McCray AT (2003). Exploring semantic groups through visual approaches. J Biomed Inform.

[18] Liu H, Bielinski SJ, Sohn S, Murphy S, Wagholikar KB, Jonnalagadda SR, Ravikumar KE, Wu ST, Kullo IJ, Chute CG (2013). An information extraction framework for cohort identification using electronic health records. AMIA Jt Summits Transl Sci Proc.

[19] Liu S, Mojarad M, Wang Y (2018). Overview of the BioCreative/OHNLP family history extraction task. ResearchGate.

[20] Shen F, Liu S, Fu S, Wang Y, Henry S, Uzuner O, Liu H (2021). Family history extraction from synthetic clinical narratives using natural language processing: overview and evaluation of a challenge data set and solutions for the 2019 National NLP Clinical Challenges (n2c2)/Open Health Natural Language Processing (OHNLP) Competition. JMIR Med Inform.

[21] South BR, Shen S, DuVall SL, Uzuner (2011). 2010 i2b2/VA challenge on concepts, assertions, and relations in clinical text. J Am Med Inform Assoc.

[22] Alsentzer E, Murphy J, Boag W (2019). Publicly available clinical BERT embeddings. arXiv.

[23] Michalopoulos G, Wang Y, Kaka H, Chen H, Wong A (2020). Umlsbert: Clinical domain knowledge augmentation of contextual embeddings using the unified medical language system metathesaurus. arXiv.

[24] Peterson KJ, Jiang G, Liu H (2020). A corpus-driven standardization framework for encoding clinical problems with HL7 FHIR. J Biomed Inform.

[25] OHNLP (2023). Rule-based family history NLP system built on lexicon. arXiv.

[26] Zhong Z, Chen D (2020). A frustratingly easy approach for entity and relation extraction. arXiv.

[27] Campos D, Matos S, Oliveira J, Sakurai S (2012). Biomedical named entity recognition: a survey of machine-learning tools. Theory and applications for advanced text mining.

