# Supplementary material for: Acquisition of a Lexicon for Family History Information: Bidirectional Encoder Representations From Transformers–Assisted Sublanguage Analysis
Source: JMIR Med Inform. 2023 Jun 27;11:e48072. doi: 10.2196/48072 (PMC10337517; doi:10.2196/48072)
Supplement: Multimedia Appendix 1 [file medinform_v11i1e48072_app1.docx]

Supplemental Table 1. Statistical comparison between original and enhanced annotations. A: No. span correction, B: No. errors removed, C: No. newly added, SNO: SNOMED codes, LIV: Living status, FDR: first degree relative, SDR: second degree relative, TDR: third degree relative

|  | | **Training set (2018/2019)** | | **Testing set (2018)** | | **Testing set (2019)** | |
| --- | --- | --- | --- | --- | --- | --- | --- |
|  |  | **Original** | **Enhanced (A,B,C)** | **Original** | **Enhanced (A,B,C)** | **Original** | **Enhanced (A,B,C)** |
| Document | | 99 | 99 | 50 | 50 | 117 | 117 |
| Chains | | 651 | 777 | 280 | 337 | 631 | 753 |
| Pairs (FM - OBS) | | 739 | 793 | 324 | 352 | 755 | 772 |
| Pairs (FM – LIV) | | 376 | 357 | 161 | 153 | 349 | 358 |
| Age | | 756 | 793 (27,16,53) | 289 | 305 (7,1,16) | 667 | 694 (4,16,42) |
| Living Status | | 415 | 437 (27,2,22) | 181 | 200 (2,1,19) | 391 | 423 (2,11,42) |
| FM | FDR | 802 | 448 | 331 | 181 | 760 | 425 |
|  | SDR |  | 337 |  | 144 |  | 320 |
|  | TDR |  | 65 |  | 29 |  | 82 |
| OBS | Entities | 978 | 1020 (68,14,55) | 465 | 488 (27,7,29) | 1062 | 1109 (20, 21, 68) |
|  | SNO (unique) | - | 968 (584) | - | 383 (193) | - | 965 (453) |
