# Supplementary material for: Acquisition of a Lexicon for Family History Information: Bidirectional Encoder Representations From Transformers–Assisted Sublanguage Analysis
Source: JMIR Med Inform. 2023 Jun 27;11:e48072. doi: 10.2196/48072 (PMC10337517; doi:10.2196/48072)
Supplement: Multimedia Appendix 2 [file medinform_v11i1e48072_app2.docx]

Supplemental Table 2. Degree of consanguinity

| Consanguinity | Members |
| --- | --- |
| First degree relative | father, mother, parent, brother, sister, sibling, child, son, daughter or any mention of “first degree relative”. |
| Second degree relative | include grandfather, grandmother, grandparent, grandson, granddaughter, grandchild, uncle, aunt, nephew, niece, half brother, half sister, half sibling or any mention of “second degree relative”. |
| Third degree relative | great grandfather, great grandmother, great grandparent, great grandson, great granddaughter, great grandchild, great uncle, great aunt, grand nephew, grand niece, cousin or any mention of “third degree relative”. |
