# Supplementary material for: Acquisition of a Lexicon for Family History Information: Bidirectional Encoder Representations From Transformers–Assisted Sublanguage Analysis
Source: JMIR Med Inform. 2023 Jun 27;11:e48072. doi: 10.2196/48072 (PMC10337517; doi:10.2196/48072)
Supplement: Multimedia Appendix 3 [file medinform_v11i1e48072_app3.docx]

Supplemental Table 3. **Performance of various BERT models fine-tuned on different datasets**

| **Model** | **Datasets** | **Precision** | **Recall** | **F1** | **No. observation for evaluation** |
| --- | --- | --- | --- | --- | --- |
| Bio_ClinicalBERT | Biocreative | 0.790 | 0.863 | 0.825 | 183 |
|  | I2B2 | 0.80 | 0.902 | 0.848 | 204 |
|  | BioCreative +I2B2 | 0.958 | 0.967 | **0.962** | 397 |
| UmlsBERT | Biocreative | 0.805 | 0.880 | 0.841 | 183 |
|  | I2B2 | 0.828 | 0.880 | 0.854 | 192 |
|  | BioCreative +I2B2 | 0.943 | 0.958 | 0.950 | 400 |
| [bert-base-uncased](https://huggingface.co/bert-base-uncased) | Biocreative | 0.813 | 0.894 | 0.852 | 180 |
|  | I2B2 | 0.826 | 0.877 | 0.851 | 195 |
|  | BioCreative +I2B2 | 0.945 | 0.974 | 0.960 | 387 |
